# Willingness to pay and moral stance: The case of farm animal welfare in Germany

**DOI:** 10.1371/journal.pone.0202193

**Published:** 2018-08-14

**Authors:** Ulrich J. Frey, Frauke Pirscher

**Affiliations:** 1 Department of Energy Systems Analysis, Institute of Engineering Thermodynamics, German Aerospace Center,Stuttgart, Germany; 2 Department of Agricultural, Environmental and Food Policy, Martin Luther University-Halle Wittenberg, Halle (Saale), Germany; Universidade do Porto Instituto de Biologia Molecular e Celular, PORTUGAL

## Abstract

This study examines the impact of ethical attitude on the willingness to pay for farm animal welfare improvement in Germany. Little is known about the pluralism of moral attitudes that may exist behind farm animal welfare issues and its relationship to customers' willingness to pay for it. Via a large survey (n = 1334) we are able to identify different moral dimensions by employing validated scales. We find utilitarian alongside deontological attitudes as well as a mixture of both. Thus, presupposing a standard moral attitude is too simple. This has implications for decision-making on markets, since the implicit normative assumptions of a utilitarian position in economics has to be critically assessed. Furthermore, we asked for the willingness to pay for various aspects of farm animal welfare improvement. We find significant positive correlations between willingness to pay and environmental concern, altruism and less apathy. Measured in Euro, a higher environmental concern has the strongest effect on WTP for all five moral scales. Outliers with higher bids are willing to pay almost five times for any aspect of farm animal welfare than the rest of the sample. A more detailed analysis of outliers demonstrates that market-based approaches have restrictions in capturing certain moral values. Moreover, the motivations behind zero bids reveal that moral concerns outweigh indifference towards animal welfare by far. This has implications how policy can be designed to serve people’s demand for higher animal welfare standards. Two other findings are of interest. First, we find a very high number of people assigning an intrinsic value to animals (90%). Second, zero bids and outlier treatment in WTP-studies deserves more careful consideration, since WTP-estimates are easily skewed by excluding these groups.

## Introduction

During recent decades the treatment of farm animals has raised public concerns in most Western societies. An increasing number of people criticise certain farming practices and the treatment of animals in modern agriculture in general [1,2,3,4]. The unease with intensive animal husbandry can be interpreted as a shift in society’s moral attitude towards animals. In many European countries, national legislation has been changed to reflect people’s increased awareness of animal well-being. In Article 13 of the Lisbon treaty [5] animals are recognised as sentient beings. Furthermore, the EU regulates husbandry, transport, and slaughter concerning farm animal welfare (FAW) in a number of directives and regulations, e.g. [6,7,5,8,9,10].

Nevertheless, many people still view the current legal standards as insufficient. Although a multitude of governance instruments exists (see [11], p. 211–260), there are two policy options that dominate the societal debate: the strengthening of statutory welfare requirements or improving FAW via market-based instruments like animal welfare labelling. While in the first case the welfare of all farm animals will be improved, the improvement in the second case depends on the willingness to pay of the consumers. Presently, we observe a strong tendency towards market-based instruments since these fit well with the currently dominating neoliberal concept of smart regulation [12,13]. A large number of national labelling schemes and market-led initiatives have been established to provide food with animal welfare standards above the legal requirements. Examples include Freedom Food, Better Leven, Bruder Hahn Initiative, Stjernekød, and Label Rouge.

However, governing animal welfare via market-based instruments implicitly assumes that the moral question on how to deal with farm animals can be framed as a question of economic decision-making. The commodification of moral concerns allows to change the living conditions of farm animals by choosing the right shopping option. Here, FAW becomes a quality attribute of the consumed animal product and the label serves to overcome the consumers’ information deficit regarding FAW. The observation that certain groups of consumers are willing and able to exercise citizenship on markets has raised the question of the motivation behind such a behaviour: Do people still act as self-oriented consumers or really as political citizens when buying products with a higher FAW standard?

According to neoclassical economics, consumers act rational on markets if they choose those bundles of commodities that maximize their individual utility. This means that an anthropocentric utilitarian perspective is assumed [14,15]. Within this narrow understanding of rational choice it is assumed that humans only care about animalsʼ well-being because the welfare of animals directly affects their own utility. Some economic studies remain within this interpretation of demand for products with higher FAW standards [16,17,18]. However, interdisciplinary studies on ethical consumption and consumer behavior on moral markets provide additional explanations why people buy products that improve the well-being of others [19,20,21,22]. Here, the compliance with societal norms has been identified as an important motivation: Internalized moral norms can lead to the feeling of a moral obligation to conduct certain non-egoistic behaviour [23]. That means that the anthropocentric utilitarian argument is only one of many moral arguments in order to treat animals well and probably not the dominating one.

Other possible ethical positions have in common that they consider non-humans as entities with an intrinsic value, but not purely instrumental to humans (e.g. [24]). Under this non-anthropocentric perspective one can subsume biocentric (all living beings have an intrinsic value), pathocentric (all sentient beings have an intrinsic value) and ecocentric attitudes (the ecosphere has an intrinsic value) [25,26].

Alongside the anthropocentric / ecocentric dichotomy, three other moral dimensions have been discussed intensively in theoretical and empirical ethics: utilitarianism vs. deontology [27,28], altruism vs. egoism [29] and apathy [30]. Therefore, we cannot assume that there is *one* concern for others, but that personal norms can be rooted in different ethical concepts which in certain aspects depart from the neoclassical paradigm of rational choice [31]. They differ in regard to the entities they assign intrinsic values (anthropocentric, pathocentric, biocentric, ecocentric) but also with regard to the commensurability of associated values.

While utilitarianism assumes trade-offs between different values, deontological ethics stipulates that some values are incommensurable or at least hierarchically ordered [32,33,34]. For the latter moral position, a person chooses an option even if it reduces his or her personal welfare exactly because this person is convinced that acting in a particular way is morally right [35]. Here it is the morality of the action, not the result of the action that determines what is morally right or wrong.

If a substantial number of individuals in a society holds deontological beliefs that deviate from key normative assumption of markets the question arises how people with such attitudes behave on a market for animal welfare products. Do people conflate their moral concerns with consumer preferences or not?

Although there is an increasing number of both economics and ethics literature dealing with different aspects of the complex issue of animal welfare (for an overview see [36,37]) only few studies combine ethical concepts with the empirical analysis of behaviour of consumers on moral markets [38,39,40,41,42,43,23]. More specifically, research at the interface between ethics and willingness to pay (WTP) in FAW is missing (but see [44]).

The majority of economic research on FAW has addressed effects of change of consumers’ WTP in animal production, transportation, slaughtering systems or breeding [45,46,47,48,37,49,50].

Although many authors point out that people’s moral attitude, too, might be part of the WTP for animal welfare improvement, it is only rarely taken explicitly into account as an explanatory variable for WTP. Besides, a further differentiation between different ethical positions is missing. If "ethics" is mentioned at all, it is used as a synonym for any kind of altruism in contrast to egoistic or selfish behaviour [36,51,52].

An exception is the work of Bennett et al. [44] who indeed consider moral attitudes as an explanatory variable for WTP on FAW but remain within the framework of an utilitarian ethics by distinguishing between different degrees of moral intensity but not between different moral stances. Thus, fundamental differences in ethical positions are neglected. In general, it is surprising that WTP analysis on FAW does not consider ethical attitude as an explanatory variable, because WTP methods as such allow for capturing different motivations for WTP. Moreover, studies in the field of environmental economics show that willingness to pay for environmental aspects has a distinct moral component [53,54,55,56,57,58,59].

Against this background, we combine insights from empirical ethics with the economic analysis of consumer behavior by considering the influence of value pluralism for the willingness to pay. Our leading questions are: To what degree are different moral attitudes reflected in market decisions? Do different ethical stances lead to a higher or lower willingness to pay? Are those who do not pay for animal welfare not interested in the living conditions of farm animals or is their moral attitude incompatible with the normative assumption of a market approach? If this is true, is this frame incompatible with moral diversity?

If the assumption of a single standard moral attitude does not hold, an analysis of a variety of moral positions might add insights to previous WTP analyses. In particular, the treatment and interpretation of zero bids and outliers would have to be reconsidered. A simple exclusion might well distort results since such expressions of WTP might be either moral protests, protests against the market assumption of trade-offs between values or protest against the value articulating vehicle [60].

### Hypotheses

The following three hypotheses were tested:

Hypothesis 1: Contrary to neo-classic model assumptions of a uniform anthropocentric utilitarian position, consumers show a range of different moral values.

Studies in empirical economics assume a single human value system. Within this system, better animal welfare is interpreted as consumer preferences for not causing damage to animals which have not yet been properly internalised into the market for animal products [18,61,62,63,51]. Thus, our first hypothesis tests whether this presupposed moral uniformity can be confirmed or whether instead moral diversity exists. In addition, it is an open empirical question whether ethical positions are mutually exclusive, which is a common assumption in the philosophical literature. Therefore, we analyse this aspect as well.

Hypothesis 2: Different moral attitudes lead to different WTP for animal welfare improvement.

While many studies dismiss moral values as not relevant for market-based decision-making, some other research has found strong effects on the willingness to pay [53,41,64]. All studies mentioned are concerned about the influence of ethical attitudes on the WTP of environmental amenities. For these environmental issues, it has been demonstrated that a higher ecocentric moral stance leads to a higher WTP [30]. If we understand an ecocentric attitude in accordance with Taylor [26] as a general respect for all living beings, farm animals are included in this group of moral entities. A higher ecocentric moral attitude thus may also translate into a higher WTP for FAW improvement.

The opposite may be true for a deontological position. Deontological attitudes not only reject the possibility of total commensurability, but also the idea of choice as a trade-off between different options [32]. Thus, we assume that a deontological position will lead–at least in some cases–to a rejection of the idea of paying for animal welfare as such. Therefore, a more deontological moral attitude should lead to a lower WTP for animal welfare improvement and, vice versa, a more utilitarian attitude to a higher WTP.

We further assume that a more altruistic attitude leads to a higher WTP for FAW improvement, if we understand altruism as an increase in utility for others [64]. This remains within the general assumption of utility maximisation and thus differs from a deontological perspective. This is what Sen [35] calls 'sympathy'.

A lack of interest for animal welfare issues should also lead to a lower WTP. Conceptualised as apathy in environmental studies, this trait has been linked to little engagement in environmental organisations [30]. Traits like apathy, environmental concern or a certain moral attitude like a more utilitarian stance are operationalized via scales, i.e. validated instruments that capture them in a precisely defined quantitative way. The scales are described in the Methods below and the SI.

In the light of these considerations, we test the general hypothesis whether different moral attitudes lead to different WTP for FAW improvement by breaking it down to the following sub-hypothesis:

2a. A higher score on the environmental scale leads to a higher WTP for FAW improvement.
2b. A lower score on the deontological scale leads to a higher WTP for FAW improvement.
2c. A higher score on the utilitarian scale leads to a higher WTP for FAW improvement.
2d. A higher score on the altruism scale leads to a higher WTP for FAW improvement.
2e. A lower score on the apathy scale leads to a higher WTP for FAW improvement.

Hypothesis 3: Market-based instruments cannot capture certain moral values.

If certain assumptions of moral attitudes are contrary to key assumptions of market behaviour, we assume that this may conflict with the idea of indicating one’s moral stance via WTP. Respondents might reject the idea of solving moral issues by consumer decisions and thus reject the market as a governance structure for societal decision making as such. This would be in line with previous arguments [65,66,15,67]. This means that individuals do not relate ethical questions to decisions in markets and thus do not feel responsible as consumers as they do as citizens. This is especially applicable for the case of animal welfare [68]. In such a case, not willing to pay would not automatically mean having no preference for an animal welfare improvement. It could also mean to refuse the governance structure of a market.

## Methods

Given that there were no known risks associated with this research study, participants of the survey and the interviews were not a vulnerable group of people, and complete confidentiality was guaranteed, we saw no need for formal ethical review before the study began. Questionnaires were completed anonymously: no information was collected that could be used to identify participants. Participants knew that they were involved in a scientific experiment, and asked for their consent to use the data. None of the participants expressed discomfort or asked to withdraw their data from the study.

### Willingness to pay

In our WTP analysis respondents were asked for the maximum amount they would be willing to pay for a certain scenario, in this case the improvement of FAW. The concept is based on the Hicksian compensation variation [69]. For animal welfare improvements, we chose four that are currently under public debate in Germany. Each scenario corresponds to a WTP question. The first question asked participants about their WTP for eggs if male chicks were not killed (all questions can be found in the S1 text). The second question was about the WTP for more space for pigs, the third one asked about the WTP for pain medication for the castration of piglets and the fourth one about the WTP for more space for laying hens. The questions covered key aspects of ethical concerns: the killing of animals, their physical suffering and the expected improvement of their well-being, in this case more space. For all aspects there are examples where more FAW improvements have been implemented (Bruderhahn Initiative sells eggs with an extra price paid for the fattening of the males; the label "Für mehr Tierschutz" and organic labels offer animal products where the animals have more space and an anaesthesia during castration in case of pigs).

Here is the text for the questions:

1. “To produce eggs only female animals are needed. Male chicks are therefore killed on their first day for economic reasons. At the moment six eggs produced on deep litter farming cost 1.32 Euro. How much more would you pay for six eggs if male chicks could be raised as broilers (in Eurocent)?”
2. In accordance with the German animal protection law the minimum space for fattening pigs is, depending on their weight, between 0.5 and 1 m^2^ space. Animal rights activists demand more space. At the moment, a chop of meat from pigs (1 kg) costs around 4.95 Euro. How much more would you pay for 1 kg of pork if pigs were accorded 1 m^2^ more space (in Eurocent)?
3. Uncastrated male fattened pigs may develop a boar smell which renders such pork unsellable. Therefore male piglets are allowed to be castrated during the first seven days after birth without anaesthesia. Castration with anaesthesia is more expensive and therefore often skipped for economic reasons. At the moment, a chop of meat from pigs (1 kg) costs around 4.95 Euro. How much more would you pay for 1 kg of pork if anaesthesia was applied to male piglets when castrated (in Eurocent)?
4. In accordance with the German animal protection law laying hens are allowed to have a space of 27 x 30 cm, which is a bit less than 1 1 /2 DIN A4 sheets. Animal rights activists demand more space. At the moment six eggs produced on deep litter farming cost 1.32 Euro. How much more would you pay for six eggs if each laying hen were accorded around 300 cm^2^ more space (in Eurocent)?

These currently debated issues and the existence of empirical examples for animal welfare improvement ensured a certain familiarity of the respondents with animal welfare problems in question. Asking the WTP for the same animal welfare improvement but for different animals allowed excluding animal specific sympathies or antipathies. We were aware that this operationalization reduces the complexity of animal welfare to four narrow aspects. However, this was necessary to elicit comparable answers.

As payment vehicle we chose open-ended questions. Although payment vehicle bias could be an issue [70], we chose this and not a payment card format, because the open-ended nature of questions is more suited to identify protesters among respondents who refuse to pay [69,71]. Other formats introduce their own respective biases. For example, surveys with discrete choice format result in a higher WTP [72,73].

Yet, the vignette describing the questions resembles payment card format, since we provided the typical price of the product in question to avoid uninformed answers [74]. The key to avoid payment vehicle bias is not so much the format per se, but the familiarity of respondents with the product and situation [70]. In our case the scenario is about buying eggs and meat at a supermarket. Therefore, we assume a very high familiarity, thus minimizing possible bias. This can be backed up by the fact that not a single one out of 1334 participants mentioned unfamiliarity with the payment vehicle in the debriefing answers or that it was inappropriate per se (see also S1 Table, reason 11).

We are aware that people's willingness to pay is often overestimated through the hypothetical nature of the questions leading to considerable upward-bias [75,76].

Since there was a separate WTP question for each welfare improvement, it was necessary to remind the participants of their budget constraint. Therefore, we added a “cheap-talk” script to reduce hypothetical bias (as suggested e.g. by [77]; see the S2 Text).

### Survey

We conducted a survey on farm animal welfare and WTP both online and offline from the 15^th^ of December 2015 to the 19^th^ of February 2016 in Halle, Germany. Three rounds of pre-tests ensured that questions were without ambiguity. The online and offline version were identical. The offline version–filling out a printed form–was conducted in several public locations (e.g. city registry office) and made up only 4.2% (69 participants) of the total sample. The majority of respondents filled out an online version (1591 participants or 95.8%) and were contacted by a university mailing list.

A randomizing mechanism in the survey software Limesurvey 2.06 (https://www.limesurvey.org/en/) assigned participants to two treatments. The offline version was randomized as well. The only difference between the two treatments was the visibility of the justification for the WTP answers–either it was visible all the time or it was only visible if the WTP entered was zero or 40% above the usual price mentioned in the text. Since there was no statistically significant effect of whether or not justifications were always visible (two-sided t-test, n.s), the data were pooled for all analyses. The analyses were conducted with R 3.2.3 [78].

To avoid any bias, the topic animal welfare was neither mentioned in the title nor in the introduction. Out of 2672 participants, 1660 (62.1%) completed the survey. The remaining 1334 participants were included in later analyses.

The survey consisted of five sections: (1) demographics, (2) WTP questions on aspects of animal welfare, (3) questions on the environmental attitude, including the scale for General Awareness of Consequences (GAC, see [79],[80]) and scales measuring moral values, e.g. altruistic tendencies and environmental apathy [30]. The fourth section (4) inquired about other aspects of animal welfare. The final section (5) employed a validated scale on deontological and utilitarian values [81]. Taken together, there were 37 questions that took around 20–30 minutes to answer. In order to be consistent, answers to almost all questions and matrices were in a 5-item-scale format, ranging from “very important” to “not at all important” or “strongly agree” to “strongly disagree”. The SI contains the WTP-questions (S1 Text), scales (S4 Text to S7 Text) and their factor loadings (S3 Table and S4 Table).

A few typing errors were corrected, if they were obvious. For example, the year of birth 19993 was corrected to 1993. All vegetarians and vegans (n = 325) were excluded from further analysis since we are asking about the WTP for a good that they do not consume. One further person was excluded since he entered nonsensical values for the WTP like ‘999999999’. The following paragraphs add some more details about two sections of the survey.

In the first section respondents were asked to provide their demographic data in order to start with neutral questions. The second section asked participants about their WTP for four aspects of animal welfare mentioned above

For those who indicated a zero bid or a high bid (above +40% of the normal price mentioned in the text) a question about the motives for their willingness to pay followed immediately (see S1 Text for the exact wording of the questions and S1 Table for all answer options available). To cover all possible reasons we provided a very broad spectrum of eleven answers and a free text option “Other”, as we were not only interested in those who pay but also in the motives of those who deviated from conforming market behaviour (answers were partly adapted from [40,82,83]. Only one answer was allowed to force participants to decide on the most important one. All free text answers are available in the data repository.

The scales employed in the third section (GAC, deontological/utilitarian, apathy and altruism scale) were constructed out of their respective items. They were aligned in one direction (see [80]) and added to obtain an index value. Scales were normalised in order to enable comparisons between them. All questions making up the scales can be found in the S4 Text to S7 Text,.

Using validated scales ensured that the information on the ethical position of respondents was reliable in regard to deontological or utilitarian stance, and anthropocentric or ecocentric value orientation. In addition, the four WTP-questions with eleven justification answers allowed to construct a fine-grained picture of consumer behaviour for a majority of the participants of the survey who gave justifications (81.5%).

We used the widely employed GAC scale for a set of attitudinal questions with regard to the environment [79,80]. This scale has been designed to capture the environmental concern of respondents. It has been reported as even more specific and to be able to distinguish between anthropocentric (egoistic and altruistic) and ecocentric value orientation [53]. However, other studies could not reproduce the factor loadings e.g. [84] that lead to these aspects; neither can we (see S4 Table for factor loadings). However, the GAC-scale is very robust as a measure for environmental concern. Since we are not aware of any validated scale specific for animal welfare, we use the GAC-scale with its focus on attitudes towards more general environmental concern aspects.

In our study, all individual Cronbachʼs alpha coefficients for the GAC-scale were greater than 0.8 and the overall scale Cronbachʼs alpha is 0.83. These coefficients replicate previous results perfectly [84]. Additionally, a comparative study has shown it to be the most robust scale for measuring environmental concern with regard to demographic effects [85]. To compensate for the lack of a third dimension (apathy) in this scale further three questions that measure apathy were added to the survey since apathy has been suggested as another important moral dimension [30].

The fourth section contained questions about various aspects of animal welfare and included a short altruism section.

The fifth and last section contained a further validated scale–the Robinson deontological / utilitarian scale [81]. This scale allowed us to reliably distinguish between individuals who make moral judgments based on either deontological or utilitarian grounds. It has been tested with a large number of participants and no item used has a factor loading below 0.57 (see S5 Table).

Due to time constraints for participants, only selected questions of scales for the dimensions of apathy [30] and altruism [86] have been chosen. The list of questions can be found in the SI, section 6.

## Results

Descriptive statistics for the demographic data of the 1334 respondents, the four willingness to pay variables and the scales used to capture moral attitudes are displayed in Table 1.

**Table 1:** Descriptive statistics of key variables in the survey (n = 1334).

Variable names	Minimum	Maximum	Mean	Median	SD
Age	16	77	32.57	28	12.13
WTP kill male chicks	0	3000	105.21	70	142.83
WTP space for pigs	0	10000	298.65	200	570.52
WTP castration pigs	0	10000	263.5	100	505.78
WTP space laying hens	0	2500	109.31	70	149.57
Apathy index	-0.87	4.7	0	-0.41	1
GAC index	-5.25	1.28	0	0.19	1
Altruism index	-4.74	1.26	0	0.06	1
Deontological index	-4.3	2.06	0	0.15	1
Utilitarianism index	-2.04	3.72	0	-0.03	1

Note: The units of the variables are as follows: Age (in years), WTP (in Eurocent), Apathy index (3–15, low values = more apathetic), GAC index (9–45, low values = less environmental concern), Altruism index (4–20, low values = less altruism), Deontological index (5–25, low values = less of a deontological position) and Utilitarian index (5–25, low values = less of a utilitarian position)

Gender is a bit skewed towards female participants (60%). More than 50% of the sample have a university degree (52.7%), while income is split relatively evenly between categories: 16% = < 500 Euro; 27% = 500–1500 Euro; 15% = 1500–2500 Euro; 12% = 2500–3500 Euro; 10% = 3500–4500 Euro; 15% = > 4500 Euro.

Representative statistical samples of demographic attributes for entire Germany indicate an average age of 44 years (http://www.bib-demografie.de), with 49% male (http://www.bpb.de) and 2716 Euro of mean annual income (http://de.statista.com). The differences are due to the sample being drawn from a university population.

### Hypothesis 1

Hypothesis 1: Contrary to neo-classic model assumptions of a uniform anthropocentric utilitarian position, consumers show a range of different moral values.

**Fig 1:**
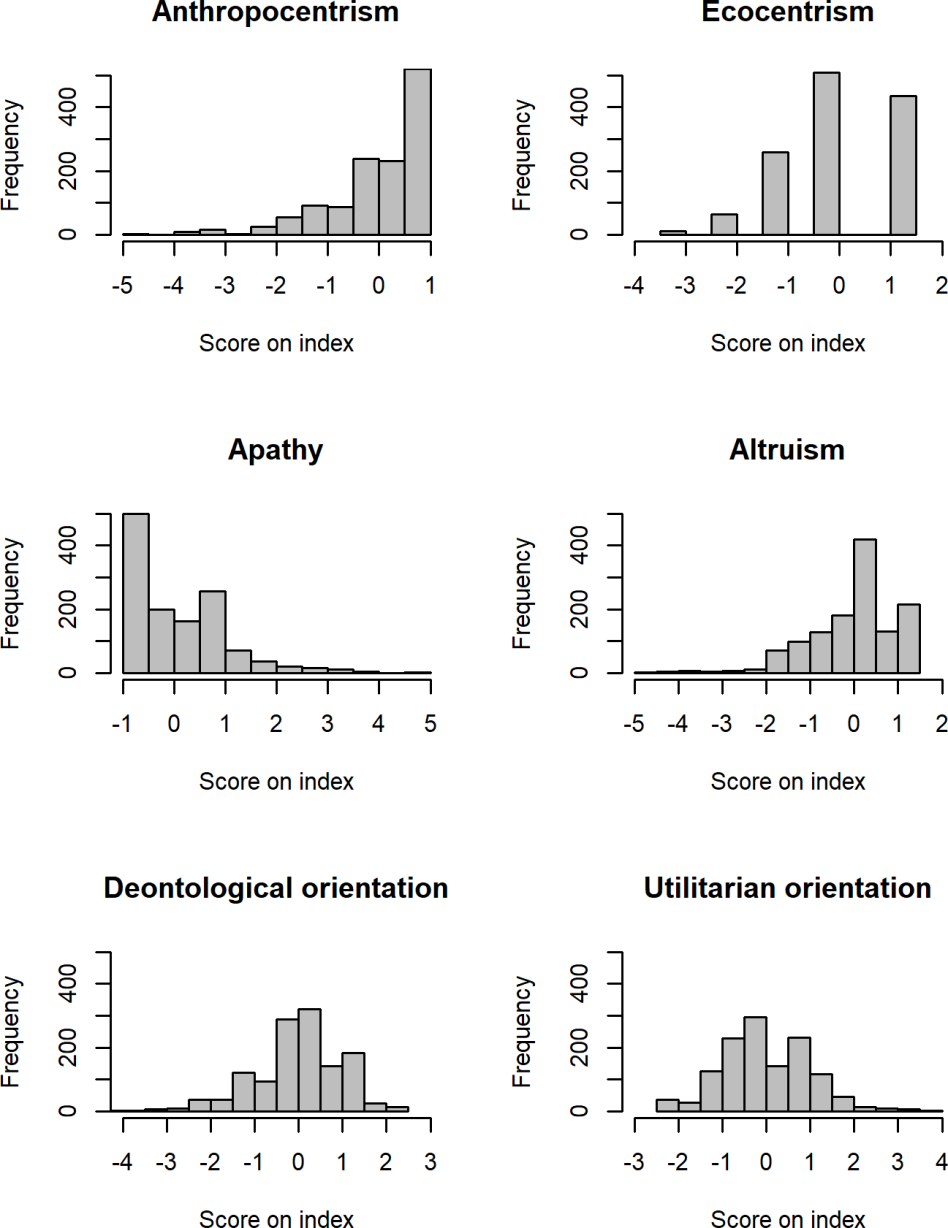
Frequencies of scores on six moral indices (anthropocentrism, ecocentrism, apathy, altruism, deontological and utilitarian orientation). Note: due to a varying number of items of the scales, the index scores possess different ranges; all indices have been scaled (mean = 0, sd = 1) for comparisons.

Our large sample clearly shows different moral attitudes in several moral “dimensions”:

Fig 1 shows that apathy and altruism are skewed: the apathy index consists of three 5-item-scale questions. Range, minimum, maximum, mean, median and standard deviation for all scales can be found in Table 1. Not surprisingly, people tend to see themselves as altruistic and not apathetic. Therefore, the distributions of answers on these two scales are presumably driven by a skewed self-perception towards perceiving oneself as more altruistic and less apathetic (higher values signify more altruism and less apathy).

Apart from the deontological / utilitarian-index, we separately checked possible moral attitudes with the question

“There are different opinions about what is morally 'good' and 'bad'. How morally good or bad an action is, depends in my opinion on: a) how good or bad the real consequences are for me b) how good or bad the real consequences are for others and society c) how good or bad the actor’s intention is d) whether rights of others are violated e) whether religious rights are violated.”

Here, only 3% of all 1334 participants opted for the “utilitarian-egoistic” option a), whereas the majority (52%) chose the “utilitarian-altruistic” answer b). 20% opted for the “deontological” answer c) and 21% for the “rights-based” answer d). Only 4% chose option e). These answers alone demonstrate a rich picture of moral attitudes.

Second, traditional philosophy perceives deontological and utilitarian attitudes as opposed and mutually exclusive [87,26]. This does not hold true for participants in our survey. The dominating moral attitude seems to be a mixture of both, since the majority (902 individuals or 68%) answer “Yes” to a question we posed independently of the deontological / utilitarian index items. We asked

“My own moral position may be described as follows: Humans and animals have certain inalienable rights (e.g. right to live) but in order to evaluate a moral action the consequences are important, too.”

Only 156 individuals (9%) answer “No” and 333 (23%) answer “Donʼt know”. This result is underscored by plotting the deontological index versus the utilitarian index (S1 Fig). If both attitudes were indeed opposed, we should be able to find two distinct clusters in the top left and bottom right corner of the figure. Instead, there is a large area of overlap.

Lastly, as mentioned above, consumer utilities in markets rest on the assumption that the consumption of animals contributes to the utility of their human consumers, thus animals have an instrumental value. When participants were asked whether humans, animals, plants and nature have an intrinsic value the majority assigned an intrinsic value to all entities. The question text explained that intrinsic means that the respective entity in question has to be considered for itself and independent of its utility for humans. The results are displayed in Table 2:

**Table 2:** Percentages of individuals assigning intrinsic values to humans, animals, plants and nature (n = 1334).

	Yes	No	Don't know
**Humans**	92	1	7
**Animals**	90	3	7
**Plants**	72	13	14
**Nature**	88	3	9

The data in Table 2 show that there is clear support for the first hypothesis. We find a number of different moral values in several dimensions. Anthropocentric utilitarianism is not the standard moral concept with regard to FAW.

### Hypothesis 2

Hypothesis 2: Different moral attitudes lead to different WTP.

An obvious follow-up step is to investigate whether the different moral positions (environmental concern, deontological and utilitarian attitude, altruism and apathy) found above result in a different WTP.

First, the correlations between moral scales and the WTP are reported in Table 3.

**Table 3:** Correlation coefficients of moral values and willingness to pay (* = 0.05, ** = 0.01, *** = 0.001, n = 1334).

	WTP killing male chicks	WTP more space for pigs	WTP pain relieve for piglet castration	WTP more space for laying hens
**Apathy-index**	-0.08	-0.07*	-0.06*	-0.11***
**Altruism-index**	0.10***	0.06*	n.s.	0.09***
**Deontological-index**	n.s.	n.s.	n.s.	n.s.
**Utilitarian-index**	0.06*	n.s.	n.s.	0.07**
**GAC**	0.13***	0.11***	0.13***	0.14***

The correlations differ somewhat across all four animal welfare issues: whereas altruism and a deontological value orientation are not correlated with the willingness to pay for FAW improvement, a lower apathy, a higher utilitarian orientation and a higher environmental awareness do indeed correspond to a small but significant increase in the willingness to pay for FAW improvement.

We were also interested in calculating the differences in the willingness to pay for these different moral orientations. Therefore, we split the sample for each index at the median. The following table (Table 4) shows the mean WTP in Euro for each group per moral dimension. For example, the first cell indicates that individuals scoring higher on the apathy-index have a mean WTP of 1 Euro more for the first WTP-question, whereas those scoring lower than the median of the apathy-index (the less apathetic individuals) have a mean willingness to pay 1.12 Euro more to prevent male chicks being killed.

**Table 4:** WTP mean in Euro per group in relation to moral scales (sample split in two groups, total n = 1334).

	Killing male chicks (Higher / Lower bids)	More space for pigs (Higher / Lower bids)	Pain relieve for piglet castration (Higher / Lower bids)	More space for laying hens (Higher / Lower bids)
**Apathy-index**	1.00 / 1.12	2.68 / 3.46	2.40 / 2.98	0.97 / 1.27
**Altruism-index**	1.18 / 0.86	3.18 / 2.68	2.77 / 2.42	1.20 / 0.93
**GAC**	1.20 / 0.89	3.44 / 2.49	3.09 / 2.13	1.27 / 0.89
**Deontolo-gical index**	1.01 / 1.09	2.99 / 2.97	2.67 / 2.59	1.08 / 1.10
**Utilitarian index**	1.12 / 0.96	3.12 / 2.82	2.78 / 2.45	1.17 / 0.99

To make these results more robust, we calculated a regression for each WTP variable (Table 5). As independent variables all demographic variables, all indices and the WTP for the other questions enter into the regression.

**Table 5:** Regression analysis for the WTP for pain relief for piglet castration (df = 1203, adjusted R-squared: 0.69).

Term	Estimate	Std.error		Statistic	P-value
(Intercept)	88.816	36.943	2.404		0.016
Age	0.977	0.869	1.124		0.261
Gender	-59.599	17.462	-3.413		0.001
Education	-3.031	3.791	-0.8		0.424
Nr kin responsible	-9.458	9.816	-0.963		0.336
Income	2.318	6.442	0.36		0.719
Altruism index	-6.986	8.486	-0.823		0.411
Apathy index	11.774	10.673	1.103		0.27
GAC index	18.627	10.926	1.705		0.088
Deontological index	-8.256	8.513	-0.97		0.332
Utilitarian index	-0.208	8.317	-0.025		0.98
WTP kill male chicks	0.321	0.074	4.346		0.000
WTP space for pigs	0.705	0.015	47.008		0.000
WTP space for laying hens	-0.012	0.072	-0.168		0.866

The regression analyses for the other three WTP variables can be found in the SI. We find that gender, the WTP for more space for pigs and the WTP to not kill male chickens have an influence on the WTP for pain relief for piglet castration.

### Hypothesis 3

Hypothesis 3: Market-based instruments cannot capture certain moral values.

The following paragraphs analyse in detail those groups that indicate a zero bid and also those who behave as outlier in the WTP analysis. The intention is to segregate those who do not want to pay because FAW is not important for them from those who behave irrationally, and those who generally reject the instrument itself.

First, a more detailed analysis of all participants and their overall motives for the WTP reveals that only 3.1 to 4.7% can be categorised as irrational because they opted for answer 4 and 6 that is "The question is too difficult / too complicated / I need more information for a decision" and "Number just invented / guessed / no special reason". A further 1.0 to 1.5% state that animal welfare is no goal for them (answer 5).

The clear majority (55 to 59%, depending on the WTP-question; see Table 2 in the SI for all percentages) of those who gave a reason for their WTP chose the answer option “Animal welfare is really important. I want to express this with my WTP. I want to contribute in a fair manner compared to others.” This demonstrates that for the majority of participants their willingness to pay in all four animal welfare issues is indeed connected to moral reasons. The second biggest group of all four questions was the option “Other”coupled with the possibility to enter a free text (14 to 21%).

One key to understand motivations behind WTP is to look more closely at the group of zero bids. Commonly, they are defined as having a WTP of zero and having provided a reason of their refusal to pay (here: zero WTP in one of the four WTP-questions and having chosen one of the answer options 2, 3, 4, 7 or 8). In this sample, 8.7% fulfil these criteria and are therefore classified as zero bids. A detailed analysis of the answer options reveals the following results: Only 2–4.7% (depending on the WTP-question) of these 171 individuals state that animal welfare is no goal for them (answer option 5) although this should be close to 100% given this is the protest group, compared to 1–1.5% in the whole sample. Instead, 16–20% choose answer option 3, which states that animal welfare is a moral question (compared to only 6.7–8.5% in the whole sample). The answer that animal welfare is important is chosen less frequently than in the whole sample (5–16% compared to 54–59%), whereas the “Other” category (the possibility to enter any free text) is chosen more frequently (22–38% vs. 14–21%). A higher percentage of zero bids sees the state as responsible (7–14% compared to 3–5%).

In addition to these analyses we take a closer look at a group that has a higher-than-average willingness to pay for FAW improvement. We suspect that a very high willingness to pay is coupled with a certain bundle of moral values different from other participants in the survey. Individuals are identified as outlier by the standard measure of 3 * interquartile range (IQR) for any WTP-question. 329 participants (24.6%) fulfil this criterion. If an outlier is defined more strictly as having a WTP of more than 3 * IQR for all four questions, this group shrinks to 63 persons (4.7%).

Concerning their assumed bundle of moral values, we find that this group of outliers indeed has different moral values, since three differences are statistically significant (Mann-Whitney-Test, n = 1334): they score higher on altruism (0.19 vs. -0.06; p < 0.001) and environmental concern (0.23 vs. 0.08; p < 0.001) and lower on apathy (-0.13 vs. 0.45, p < 0.001).

The differences between a deontological and a utilitarian value orientation are not so pronounced and thus not significant. This result is robust since both direction and statistical significance of differences in moral values do not change if this group is restricted to outliers that have a WTP of 3 * IQR in all four questions (4.7%).

Most importantly, the differences in WTP between outliers (n = 329) and all other participants (n = 1005) are massive: 2.44 vs. 0.56 Euro for not killing male chicks; 6.91 vs. 1.62 Euro for more space for pigs; 6.39 vs. 1.32 Euro for pain relieve for piglet castration and 2.48 vs. 0.60 Euro for more space for laying hens.

Another question concerns the scope and influence of market-based instruments which may not be accepted by all. Therefore, we asked whether a particular animal welfare issue (for all four WTP-questions) should be solved by individual consumer decisions or by the state. Therefore, this evidence can be used to estimate the relevance of market-based instruments in general. For all four WTP-questions the mean percentage of individuals who assign responsibility to the state is 4.8%. A further 7.5% think that FAW improvement cannot be regulated with money and 16.8% mention other reasons.

As a conclusion, the hypothesis is supported that market-based instruments have restrictions in capturing certain moral values.

## Discussion

Our results of value plurality with regard to FAW are in accordance with the only existing study on empirical ethics of FAW of Johansson-Stenman [88,40], who identified an altruistic consequentialist position as the dominating one for Swedish students, but one third that holds other fundamental ethical views like rights based ethics with regard to FAW issues. That means that the implicit normative assumptions of a utilitarian position in economics should be critically assessed, especially for decision-making on moral markets. Here, other moral perspectives can have explanatory power for human behaviour as well. The fact that the same person holds several different ethical positions at the same time supports the assumption that utilitarian and deontological stances are not mutually exclusive [89], as has been claimed both in traditional philosophy [87] and modern environmental ethics [26]. This evidence further stresses the context dependency of the moral stance. For empirical ethics this also means to critically reflect on the suitability of single-option versus multi-option answers in research design. The very high number of people assigning an intrinsic value to animals (90%) supports the results of other studies that ethical reasons become more and more important when choosing animal products, in comparison to previous decades [90].

With regard to the ongoing debate on whether moral values do influence the WTP [54] our survey (n = 1334) indicates that market decisions are indeed influenced by moral values. The evidence is mixed, however. On the one hand, correlations show relatively clear evidence for such an influence. On the other hand, all moral indices remain insignificant in the regression analyses. One reason for that may be that there is no specific validated scale for animal welfare issues and we therefore had to rely on more general indices for environmental concerns.

Besides such methodological problems, this may point to context-specific reasons and the underlying complexity of animal welfare topics. In any case, this conflicting evidence already shows that models trying to explain WTP for FAW improvement without detailed moral considerations are too simple.

Besides the influence of moral concerns to WTP we would like to point to the behaviour of zero bids and outliers. From an economical perspective, the interpretation of a zero bid is straightforward–these individuals are not willing to pay anything for animal welfare improvement. Compared to other studies, this is relatively low, but within normal range. A meta-analysis finds a mean of 18% across many designs, see [91].

However, the analysis of answer options reveals a completely different picture. A higher percentage than in the whole sample considers FAW improvement as a moral issue.

If zero bids and outlier behaviour are associated with ethical attitudes indicating high awareness for other-regarding concerns then “not paying”or paying very much can no longer be interpreted as no interest in the topic or irrationality. These findings suggest that zero bids and outlier treatment in WTP-studies deserves more careful consideration, since WTP-estimates are easily skewed by excluding these groups.

These observations have certain implications for policies. First, consumer decisions might be influenced by labelling schemes because there is a majority of consumers with a utilitarian moral stance who are concerned about FAW and are willing to pay for it (the “concerned carnivore”, as it has been called, e.g. by [62]. However, the group of morally concerned is much larger than those who pay.

Our results indicate that a certain notion of morality can lead to a rejection of the idea of framing a moral problem as an economic one. Some individuals do not feel responsible as consumers, but might accept some political actions by the government as citizens. Thus, certain ethical attitudes can be contradictory to the idea of “voting with the dollar”[92] but support a political interpretation of the animal welfare problem.

Differentiating between distinct moral stance can add a further explanation to the differences between behaviour as consumer and the attitude of a moral citizen [93,94] which research constantly has found. Not paying can also be caused by consumers' awareness of the complexity of the animal welfare issue. The open commentary fields indicate that single measures like more space for a small proportion of animals as common in labelling initiatives are refused by the majority. Instead, a “holistic” approach for “a decent existence in all respects” is desired by many.

This highlights a general conflict of WTP analysis that exists between the complexity of the animal welfare issue and the methodological need to reduce the situation of decision-making to clearly defined change in animal welfare. Environmental ethics faces the same problem and different studies chose different trade-offs between improved methodological rigidity and problematic reductionism [95,96]. With the option of an open commentary field we tried to compensate this methodological reductionism. However, animal welfare label often faces a similar conflict between the need to make various simultaneous changes in animal husbandry to improve animal welfare substantially and to develop a label that consumer associate with certain clearly defined measurements. This is represented in labelling design with multi-level labels like Better Leven and Tierwohllabel [97,63,98]. For further research it would be interesting to analyse whether different moral stances conceptualise animal welfare differently which would lead to different preferences for labelling design.

These results in connection with the observed consumer behavior clearly mark a changing attitude towards animals that has not yet found the appropriate channels to articulate itself. Market-based solutions can create awareness for animal welfare issues. They allow a certain group of consumers to shop in accordance with their value system, but they cannot capture the variety of moral stances and cannot replace a societal debate about the status animals should have in our society and how humans have to restrict their actions to respect the interest of animals. Our results on the assignment of intrinsic values to animals support this alleged change in attitude.

## Conclusions

Although FAW is one of the most prominent ethical topics in agricultural production, very little is known about the ethical attitudes that drive these moral concerns and their conversion into market behaviour. This paper has analysed several moral dimensions that have been associated with animal welfare–general environmental concern as well as attitudes, be they anthropocentric or ecocentric, apathetic, altruistic, deontological or utilitarian.

We find utilitarian alongside deontological attitudes as well as a mixture of both. Thus, presupposing a standard moral attitude is too simple, a fortiori because the possible combination of these four dimensions already indicates a plurality of moral positions.

Our findings show that people do interpret FAW improvement as a moral issue and that their moral concerns for animals are rooted in different ethical positions. Thus, it is a plurality of moral stances besides economic, psychological or social factors that determine the awareness for FAW issues and influences the behaviour on moral markets. For further research the development of a moral scale for animal ethics would be an important step. The interpretation of FAW improvement as an ethical issue is confirmed by the very high number of people assigning an intrinsic value to animals.

The correlation-analysis indicates that environmental concern, apathy and altruism influence the WTP for animal welfare. The effects are in line with previous results. However, the effects are not very pronounced and need further investigation–it is yet unclear which factors drive the results since regression analysis does not indicate any influence of them.

Measured in Euro, a higher environmental concern has the strongest effect on WTP for all five moral scales. Outliers with higher bids are willing to pay almost five times for any aspect of farm animal welfare than the rest of the sample. Since we also find some significant correlations between WTP and moral attitudes, we suggest that moral values seem to matter for market decisions, here concerning FAW improvement.

If we take value pluralism in our society seriously, we conclude that both economists and politicians need to critically reflect about the frame they implicitly choose with a market-based instrument for reacting to moral concerns on FAW.

## Supporting information

S1 Fig Deontological vs. utilitarian index. (DOC)

S1 Table Answer options for reasons for WTP-questions. (DOC)

S2 Table Percentages of answer options for reasons for 4 WTP-questions. (DOC)

S3 Table Factor loadings for the GAC-scale with three factors. (DOC)

S4 Table Factor loadings for the GAC-scale with two factors. (DOC)

S5 Table Factor loadings for the Robinson-scale with two factors. (DOC)

S1 Text Answer options for WTP. (DOC)

S2 Text Cheap talk script. (DOC)

S3 Text Framing texts. (DOC)

S4 Text Altruistic value orientation. (DOC)

S5 Text Apathic value orientation. (DOC)

S6 Text Deontological / utilitarian value orientation. (DOC)

S7 Text Anthropocentric and ecocentric value orientation. (DOC)
