# Correction: Willingness to pay and moral stance: The case of farm animal welfare in Germany

**DOI:** 10.1371/journal.pone.0205551

**Published:** 2018-10-05

**Authors:** The PLOS ONE Staff

Incorrect versions of the Supporting Information files were included in error. The publisher apologizes for this error. Please find the correct Supporting Information files here.

## Supporting information

S1 Fig Deontological vs. utilitarian index. (DOC)

S1 Table Answer options for reasons for WTP-questions. (DOC)

S2 Table Percentages of answer options for reasons for 4 WTP-questions. (DOC)

S3 Table Factor loadings for the GAC-scale with three factors. (DOC)

S4 Table Factor loadings for the GAC-scale with two factors. (DOC)

S5 Table Factor loadings for the Robinson-scale with two factors. (DOC)

S1 Text Answer options for WTP. (DOC)

S2 Text Cheap talk script. (DOC)

S3 Text Framing texts. (DOC)

S4 Text Altruistic value orientation. (DOC)

S5 Text Apathic value orientation. (DOC)

S6 Text Deontological / utilitarian value orientation. (DOC)

S7 Text Anthropocentric and ecocentric value orientation. (DOC)
