# Supplementary material for: Willingness to pay and moral stance: The case of farm animal welfare in Germany
Source: PLoS One. 2018 Aug 14;13(8):e0202193. doi: 10.1371/journal.pone.0202193 (PMC6091959; doi:10.1371/journal.pone.0202193)
Supplement: S3 Table — (DOC) [file pone.0202193.s004.doc]

|  |  |
| --- | --- |
|  |  |
|  |  |
|  |  |
|  |  |
|  |  |
|  |  |
|  |  |
|  |  |
|  |  |
|  |  |
|  |  |

|  |  |  |  |  |  |  |  |  |  |  |  |
| --- | --- | --- | --- | --- | --- | --- | --- | --- | --- | --- | --- |
|  |  |  |  |  |  |  |  |  |  |  |  |
|  |  |  |  |  |  |  |  |  |  |  |  |
|  |  |  |  |  |  |  |  |  |  |  |  |
|  |  |  |  |  |  |  |  |  |  |  |  |

# **S3 Table. Factor loadings for the GAC-scale with three factors**

| Factor 1 | Factor 2 | Factor 3 | Uniqueness |
| --- | --- | --- | --- |
| 0.416 | 0.219 | 0.298 | 0.690 |
| 0.131 | 0.188 | 0.468 | 0.729 |
| 0.154 | 0.592 | 0.199 | 0.586 |
| 0.140 | 0.646 | 0.220 | 0.515 |
| 0.251 | 0.663 | 0.115 | 0.484 |
| 0.779 | 0.223 | 0.110 | 0.331 |
| 0.834 | 0.194 | 0.184 | 0.232 |
| 0.804 | 0.187 | 0.235 | 0.264 |
| 0.387 | 0.252 | 0.408 | 0.621 |

Chi^2^-statistic, χ2 = 25.41, df = 12, p < 0.013.

Factor analysis was performed with R 3.3.1, package stats. The factor loadings for the GAC scale with three factors and a varimax rotation are as follows:

|  |  |  |
| --- | --- | --- |
|  |  |  |
|  |  |  |
|  |  |  |
|  |  |  |
|  |  |  |
|  |  |  |
|  |  |  |
|  |  |  |
|  |  |  |

|  |  |  |
| --- | --- | --- |
|  |  |  |
|  |  |  |
|  |  |  |
|  |  |  |
|  |  |  |
|  |  |  |
|  |  |  |
|  |  |  |
|  |  |  |
|  |  |  |

Chi2-statistic,
