# Supplementary material for: Willingness to pay and moral stance: The case of farm animal welfare in Germany
Source: PLoS One. 2018 Aug 14;13(8):e0202193. doi: 10.1371/journal.pone.0202193 (PMC6091959; doi:10.1371/journal.pone.0202193)
Supplement: S4 Table — (DOC) [file pone.0202193.s005.doc]

|  |  |
| --- | --- |
|  |  |
|  |  |
|  |  |
|  |  |
|  |  |
|  |  |
|  |  |
|  |  |
|  |  |
|  |  |
|  |  |

|  |  |  |  |  |  |  |  |  |  |  |  |
| --- | --- | --- | --- | --- | --- | --- | --- | --- | --- | --- | --- |
|  |  |  |  |  |  |  |  |  |  |  |  |
|  |  |  |  |  |  |  |  |  |  |  |  |
|  |  |  |  |  |  |  |  |  |  |  |  |
|  |  |  |  |  |  |  |  |  |  |  |  |

|  |  |  |  |
| --- | --- | --- | --- |
|  |  |  |  |
|  |  |  |  |
|  |  |  |  |
|  |  |  |  |
|  |  |  |  |
|  |  |  |  |
|  |  |  |  |
|  |  |  |  |
|  |  |  |  |

Chi**S4 Table. Factor loadings for the GAC-scale with two factors**

| Factor 1 | Factor 2 | Uniqueness |
| --- | --- | --- |
| 0.455 | 0.285 | 0.711 |
| 0.207 | 0.305 | 0.864 |
| 0.171 | 0.624 | 0.581 |
| 0.158 | 0.688 | 0.502 |
| 0.258 | 0.638 | 0.526 |
| 0.778 | 0.220 | 0.346 |
| 0.849 | 0.216 | 0.232 |
| 0.828 | 0.226 | 0.263 |
| 0.442 | 0.350 | 0.682 |

Chi2-statistic, χ2 = 82.14, df = 19, p < 0.001.

|  |  |  |
| --- | --- | --- |
|  |  |  |
|  |  |  |
|  |  |  |
|  |  |  |
|  |  |  |
|  |  |  |
|  |  |  |
|  |  |  |
|  |  |  |
|  |  |  |

Chi2-statistic,
