# Supplementary material for: Willingness to pay and moral stance: The case of farm animal welfare in Germany
Source: PLoS One. 2018 Aug 14;13(8):e0202193. doi: 10.1371/journal.pone.0202193 (PMC6091959; doi:10.1371/journal.pone.0202193)
Supplement: S6 Text — (DOC) [file pone.0202193.s012.doc]

|  |  |
| --- | --- |
|  |  |
|  |  |
|  |  |
|  |  |
|  |  |
|  |  |
|  |  |
|  |  |
|  |  |
|  |  |
|  |  |

|  |  |  |  |  |  |  |  |  |  |  |  |
| --- | --- | --- | --- | --- | --- | --- | --- | --- | --- | --- | --- |
|  |  |  |  |  |  |  |  |  |  |  |  |
|  |  |  |  |  |  |  |  |  |  |  |  |
|  |  |  |  |  |  |  |  |  |  |  |  |
|  |  |  |  |  |  |  |  |  |  |  |  |

|  |  |  |  |
| --- | --- | --- | --- |
|  |  |  |  |
|  |  |  |  |
|  |  |  |  |
|  |  |  |  |
|  |  |  |  |
|  |  |  |  |
|  |  |  |  |
|  |  |  |  |
|  |  |  |  |

|  |  |  |
| --- | --- | --- |
|  |  |  |
|  |  |  |
|  |  |  |
|  |  |  |
|  |  |  |
|  |  |  |
|  |  |  |
|  |  |  |
|  |  |  |

Chi

|  |  |  |
| --- | --- | --- |
|  |  |  |
|  |  |  |
|  |  |  |
|  |  |  |
|  |  |  |
|  |  |  |
|  |  |  |
|  |  |  |
|  |  |  |
|  |  |  |

Chi2-statistic, S6 Text. Deontological / utilitarian value orientation

The questions constitute the validated Robinson scale ([3]). Questions 1, 3, 5, 7, 9 constitute the deontological, questions 2, 4, 6, 8, 10 the utilitarian value orientation.

What is your opinion about the following statements:

1. It is never morally justified to cause someone harm.
2. Rules and laws are irrelevant; whether an action produces happiness is all that matters when deciding how to act.
3. If an action is a violation of society’s most basic rules it should not be committed; even if it will result in a large amount of good.
4. Rules and laws should only be followed when they maximize happiness.
5. Some aspects of humanity are sacred and should never be violated no matter the possible gain.
6. If rules and laws do not maximize happiness for people they should be ignored.
7. Some rules and laws are universal and are binding no matter the circumstances you find yourself in.
8. The only moral principle that needs to be followed is that one must maximize happiness.
9. Some rules should never be broken.
10. People that fail to maximize happiness are doing something morally wrong.

## References

3. Robinson JS (2012) The Consequentialist Scale: Elucidating the Role of Deontological and Utilitarian Beliefs in Moral Judgments. Available: http://hdl.handle.net/1807/33868.
