# Supplementary material for: Willingness to pay and moral stance: The case of farm animal welfare in Germany
Source: PLoS One. 2018 Aug 14;13(8):e0202193. doi: 10.1371/journal.pone.0202193 (PMC6091959; doi:10.1371/journal.pone.0202193)
Supplement: S7 Text — (DOC) [file pone.0202193.s013.doc]

|  |  |
| --- | --- |
|  |  |
|  |  |
|  |  |
|  |  |
|  |  |
|  |  |
|  |  |
|  |  |
|  |  |
|  |  |
|  |  |

|  |  |  |  |  |  |  |  |  |  |  |  |
| --- | --- | --- | --- | --- | --- | --- | --- | --- | --- | --- | --- |
|  |  |  |  |  |  |  |  |  |  |  |  |
|  |  |  |  |  |  |  |  |  |  |  |  |
|  |  |  |  |  |  |  |  |  |  |  |  |
|  |  |  |  |  |  |  |  |  |  |  |  |

|  |  |  |  |
| --- | --- | --- | --- |
|  |  |  |  |
|  |  |  |  |
|  |  |  |  |
|  |  |  |  |
|  |  |  |  |
|  |  |  |  |
|  |  |  |  |
|  |  |  |  |
|  |  |  |  |

|  |  |  |
| --- | --- | --- |
|  |  |  |
|  |  |  |
|  |  |  |
|  |  |  |
|  |  |  |
|  |  |  |
|  |  |  |
|  |  |  |
|  |  |  |

Chi

|  |  |  |
| --- | --- | --- |
|  |  |  |
|  |  |  |
|  |  |  |
|  |  |  |
|  |  |  |
|  |  |  |
|  |  |  |
|  |  |  |
|  |  |  |
|  |  |  |

Chi2-statistic, S7 Text. Anthropocentric and ecocentric value orientation

This scale is the GAC scale ([4]):

1. Environmental protection benefits everyone.
2. Over the next decade, thousands of species of plants and animals will become extinct.
3. Claims that we are changing the climate are greatly exaggerated.
4. While some local plants and animals may have been harmed by environmental degradation, over the whole Earth there has been little effect.
5. Environmental threats to public health have been exaggerated.
6. Environmental protection is beneficial to my health.
7. Environmental protection will provide a better world for me and my children.
8. Environmental protection will help me to have a better quality of life.
9. Environmental damage generated here harms people all over the world.

## References

4. Stern PC, Dietz T, Guagnano GA (1995) The New Ecological Paradigm in Social-Psychological Context. Environ Behav 27 (6): 723–743.
