# Supplementary figures and images for: Correction: Willingness to pay and moral stance: The case of farm animal welfare in Germany
Source: PLoS One. 2018 Oct 5;13(10):e0205551. doi: 10.1371/journal.pone.0205551 (PMC6173451; doi:10.1371/journal.pone.0205551)

**S1 Fig. Deontological vs. utilitarian index**


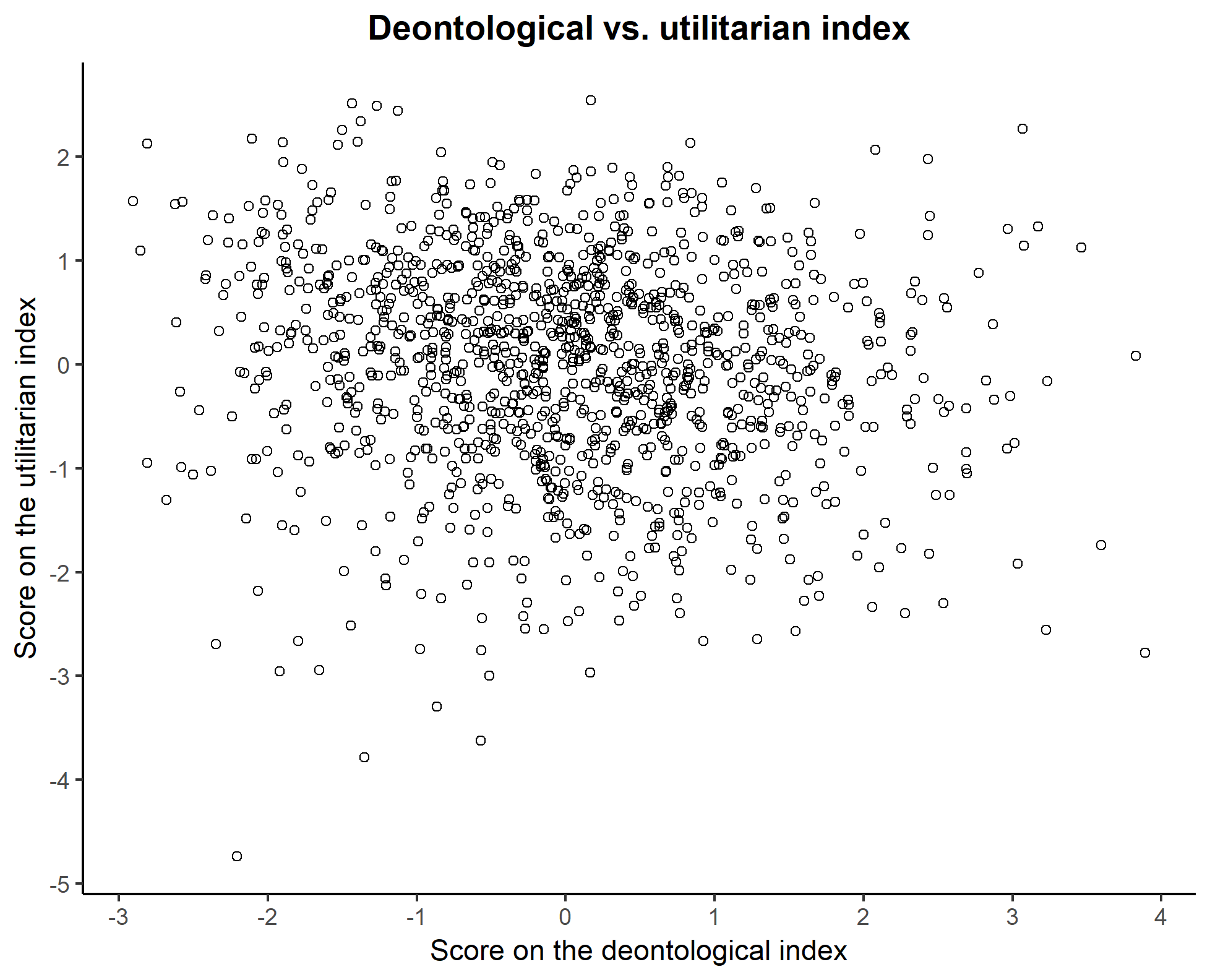

Supplement: S1 Fig — (DOC) [file pone.0205551.s001.doc]
