# Supplementary material for: Correction: Willingness to pay and moral stance: The case of farm animal welfare in Germany
Source: PLoS One. 2018 Oct 5;13(10):e0205551. doi: 10.1371/journal.pone.0205551 (PMC6173451; doi:10.1371/journal.pone.0205551)
Supplement: S1 Table — (DOC) [file pone.0205551.s002.doc]

**S1 Table. Answer options for reasons for WTP-questions**

| **Answer option for WTP** | **Answer** |
| --- | --- |
| **1** | I already pay enough for food. |
| **2** | It is unfair to ask me to pay. |
| **3** | Animal welfare is a moral question and cannot be regulated with money. |
| **4** | The question is too difficult / too complicated / I need more information for a decision. |
| **5** | Animal welfare is no goal for me. |
| **6** | Number just invented / guessed / no special reason. |
| **7** | Animal welfare problems cannot be solved by individuals. Therefore the government should deal with it (e.g. via taxes or fees), not me. |
| **8** | I can expect animal welfare and should not pay for it. |
| **9** | I already spend much for animal welfare initiatives. |
| **10** | Animal welfare is really important. I want to express this with my willingness to pay. I want to contribute in a fair manner compared to others. |
| **11** | Other: free text entry |
