# Supplementary material for: Correction: Willingness to pay and moral stance: The case of farm animal welfare in Germany
Source: PLoS One. 2018 Oct 5;13(10):e0205551. doi: 10.1371/journal.pone.0205551 (PMC6173451; doi:10.1371/journal.pone.0205551)
Supplement: S2 Table — (DOC) [file pone.0205551.s003.doc]

**S2 Table. Percentages of answer options for reasons for 4 WTP-questions**

|  | **1** | **2** | **3** | **4** | **5** | **6** | **7** | **8** | **9** | **10** | **11** |
| --- | --- | --- | --- | --- | --- | --- | --- | --- | --- | --- | --- |
| **WTP killing male chickens** | 2 | 0.2 | 8.4 | 4.5 | 1.4 | 3.6 | 4.4 | 2 | 0.2 | 58.8 | 14.5 |
| **WTP more space for pigs** | 2 | 0.2 | 7.2 | 3.1 | 1 | 4.2 | 5 | 1.7 | 0.3 | 54.8 | 20.6 |
| **WTP pain reliever for piglet castration** | 1.9 | 0.4 | 8.5 | 4.7 | 1.5 | 4 | 4.4 | 2.7 | 0.5 | 55.8 | 15.6 |
| **WTP more space for chickens** | 2.5 | 0.2 | 6.7 | 3.5 | 1.3 | 4.6 | 5.3 | 2.4 | 0.5 | 58.8 | 14.2 |

For the corresponding text to the answer options in S2 Table, see S1 Table.
