# Supplementary material for: Correction: Willingness to pay and moral stance: The case of farm animal welfare in Germany
Source: PLoS One. 2018 Oct 5;13(10):e0205551. doi: 10.1371/journal.pone.0205551 (PMC6173451; doi:10.1371/journal.pone.0205551)
Supplement: S5 Table — (DOC) [file pone.0205551.s006.doc]

**S5 Table. Factor loadings for the Robinson-scale with two factors**

| Factor 1 | Factor 2 | Uniqueness |
| --- | --- | --- |
| 0.185 | 0.222 | 0.917 |
| -0.183 | 0.272 | 0.893 |
|  | 0.510 | 0.739 |
| -0.144 | 0.663 | 0.540 |
|  | 0.662 | 0.553 |
| 0.529 | -0.171 | 0.691 |
| 0.714 | -0.145 | 0.470 |
| 0.628 | -0.165 | 0.578 |
| 0.547 |  | 0.700 |
| 0.469 |  | 0.777 |

Chi2-statistic, χ2 = 232.11, df = 26, p < 0.001.
