# Supplementary material for: Correction: Willingness to pay and moral stance: The case of farm animal welfare in Germany
Source: PLoS One. 2018 Oct 5;13(10):e0205551. doi: 10.1371/journal.pone.0205551 (PMC6173451; doi:10.1371/journal.pone.0205551)
Supplement: S1 Text — (DOC) [file pone.0205551.s007.doc]

# S1 Text. Answer options for WTP

Wording for the Willingness to pay-questions:

1. To produce eggs only female animals are needed. Male chickens are therefore killed on their first day for economic reasons. At the moment six eggs produced on deep litter farming cost 1.32 Euro. How much more would you pay for six eggs if male chickens could be raised as mast animals (in Eurocent)?
2. In accordance with the German animal protection law the minimum space for fattening pigs is, depending on their weight, between 0.5 and 1 m² space. Animal rights activists demand more space. At the moment, a chop of meat from pigs (1 kg) costs around 4.95 Euro. How much more would you pay for 1 kg of pork if pigs were accorded 1 m² more space (in Eurocent)?
3. Uncastrated male fattened pigs may develop a boar smell which renders such pork unsellable. Therefore male piglets are allowed to be castrated during the first seven days after birth without anaesthesia. Castration with anaesthesia is more expensive and therefore often skipped for economic reasons. At the moment, a chop of meat from pigs (1 kg) costs around 4.95 Euro. How much more would you pay for 1 kg of pork if anaesthesia was applied to male piglets when castrated (in Eurocent)?
4. In accordance with the German animal protection law laying hens are allowed to have a space of 27 x 30 cm, which is a bit less than 1 1 /2 DIN A4 sheets. Animal rights activists demand more space. At the moment six eggs produced on deep litter farming cost 1.32 Euro. How much more would you pay for six eggs if each laying hen were accorded around 300 cm² more space (in Eurocent)?
