# Supplementary material for: Correction: Willingness to pay and moral stance: The case of farm animal welfare in Germany
Source: PLoS One. 2018 Oct 5;13(10):e0205551. doi: 10.1371/journal.pone.0205551 (PMC6173451; doi:10.1371/journal.pone.0205551)
Supplement: S2 Text — (DOC) [file pone.0205551.s008.doc]

S2 Text: Cheap Talk Script

Please have in mind that you have a limited budget at your disposal.
