# Supplementary material for: Correction: Willingness to pay and moral stance: The case of farm animal welfare in Germany
Source: PLoS One. 2018 Oct 5;13(10):e0205551. doi: 10.1371/journal.pone.0205551 (PMC6173451; doi:10.1371/journal.pone.0205551)
Supplement: S3 Text — (DOC) [file pone.0205551.s009.doc]

Supporting Information

# S3 Text. Framing texts

## Text treatment 1

Please imagine that you are standing in your supermarket, where you usually buy food. After passing several rows of products you are now standing in front of the eggs shelve. You have the choice between two sorts of eggs. You do not see any difference except the price (1.32 and 1.42) and the types of housing (deep litter or free range). You know that these eggs do not differ in taste or in their effects on your health. Which eggs do you buy?

## Text treatment 2

Please imagine that while driving through the country you saw a sign for a farm selling eggs. Now you are standing on the farm. You can see a stable with a neighbouring meadow where a small number of laying hens run around freely. Behind, there is a large stable where several hundreds of laying hens sit densely packed on the ground. In the middle of the stable there is a conveyor belt which automatically removes the excrements of the animals. However, the litter is wet at several places. You see pecking fights and some injured animals. You go into the farm shop. Here, you can buy eggs of the deep litter housing (the stable) as well as free range eggs. The prices are 1.32 for deep litter eggs and 1.42 for free range eggs. Which eggs do you buy?
