# Supplementary material for: Correction: Willingness to pay and moral stance: The case of farm animal welfare in Germany
Source: PLoS One. 2018 Oct 5;13(10):e0205551. doi: 10.1371/journal.pone.0205551 (PMC6173451; doi:10.1371/journal.pone.0205551)
Supplement: S4 Text — (DOC) [file pone.0205551.s010.doc]

## S4 Text. Altruistic value orientation

The items are taken from the „Helping attitude scale (HAS)“ ([1]):

1. If a person in front of me at the supermarket checkout has a few cents too little, I pay the difference.
2. I feel good when I can help someone else.
3. It is an important goal of education to teach children to help others.
4. I help others even if they are strangers to me.

## References

1. Nickell GS (1998) The Helping Attitude Scale: A new measure of prosocial tendencies. Available: http://web.mnstate.edu/nickell/HAS20%20Plus%20Scoring%20and%20Citations.doc.
