# Supplementary material for: Correction: Willingness to pay and moral stance: The case of farm animal welfare in Germany
Source: PLoS One. 2018 Oct 5;13(10):e0205551. doi: 10.1371/journal.pone.0205551 (PMC6173451; doi:10.1371/journal.pone.0205551)
Supplement: S5 Text — (DOC) [file pone.0205551.s011.doc]

## S5 Text. Apathic value orientation

This scale is adapted from ([2]):

1. Environmental threats such as deforestation and climate warming have been exaggerated.
2. Given enough time, most environmental problems will solve themselves.
3. Too much emphasis has been placed on conservation.

## References

2. Gagnon Thompson SC, Barton MA (1994) Ecocentric and anthropocentric attitudes toward the environment. J Environ Psychol 14 (2): 149–157.
